# Effects of lyophilized *Lactiplantibacillus plantarum* DC2 probiotic powder on gastrointestinal morphology and microbiota in mice

**DOI:** 10.3389/fmicb.2026.1858099

**Published:** 2026-09-28

**Authors:** Juzuo Zhang, Jun Jiang, Mingyu Wang, Demiao Zeng, Junfei Xu

**Affiliations:** 1 Wuling Mountain Food Engineer Collaborative Innovation Center of Hunan Province/Key Laboratory of Research and Utilization of Ethnomedicinal Plant Resources of Hunan Province/Hunan Provincial Higher Education Key Laboratory of Intensive Processing Research on Mountain Ecological Food, College of Biological and Food Engineering, Huaihua University, Huaihua, China; 2 Joint Surgery Department, Southern Central Hospital of Yunnan Province/First People's Hospital of Honghe State, Honghe, China

**Keywords:** gut microbiota, intestinal morphology, *Lactiplantibacillus plantarum*, lyophilized probiotic powder, lyoprotectant, process optimization

## Abstract

Probiotics have potential benefits in modulating gastrointestinal function. However, their efficacy and application depend largely on specific bacterial strains and environmental contexts. In our previous study, a strain of *Lactiplantibacillus plantarum* (*L. plantarum*) DC2 exhibited superior functionality in pork fermentation processes and was granted a Chinese patent (ZL202110743486.8). However, its storage and application characteristics remain unclear. To evaluate its direct application potential for gastrointestinal regulation, in this study, the formula and process of lyophilized *L. plantarum* DC2 powder were optimized via single-factor design (SFD) and response surface methodology (RSM). The optimized protectant consisted of fat-free milk powder, trehalose, glycerine, and L-sodium glutamate. The survival rate and storage stability of lyophilized bacterial powder were maintained at optimal level under optimized conditions. Intragastric administration experiments revealed that the abundance of *Lactobacillus* in both the stomach and jejunum significantly increased, accompanied by shifts in intestinal morphological features and modulation of the intestinal microbial composition and abundance. In conclusion, a reliable lyophilization protocol was established for optimal survival rate and storage stability of lyophilized *L. plantarum* DC2 powder, which was capable of modulating intestinal morphology and gut microbial profiles in mice. This study lays a foundation for its further development as a probiotic candidate.

## Introduction

Probiotics have garnered significant interest across the food science, biotechnology, and clinical medicine fields because of their potential applications in promoting gastrointestinal health and managing chronic diseases (Sarita et al., 2024; Williams, 2010; Al-Madhagi and Alramo, 2024). An increasing number of microbial species with probiotic properties have been investigated, primarily bacteria [*Lactobacillus* (Xu et al., 2024b), *Bifidobacteria* (Wu et al., 2023) and *Bacillus* (Cutting, 2011; Payne et al., 2024)] and fungi [*Saccharomyces* (Cui et al., 2022), *Aspergillus* (Wang et al., 2023) and *Mucor* (Heo et al., 2023)]. Among the diverse probiotic microorganisms, *Lactobacillus* species are particularly notable for their roles in fermented food production and therapeutic interventions, with more than 261 identified species exhibiting functional benefits (Xu et al., 2024b). These Gram-positive bacteria that can regulate the intestinal microbiota, enhance immune responses, and alleviate obesity, gout and inflammatory bowel disease have been extensively studied (Sarita et al., 2024; Dempsey and Corr, 2022). However, strain-specific variations in functional properties necessitate rigorous evaluation of each candidate probiotic through *in vitro* and *in vivo* studies before clinical or industrial application.

Our research group previously isolated *Lactiplantibacillus plantarum* (*L. plantarum*) DC2 from traditional fermented foods; this strain displayed cholesterol-lowering activity and was successfully utilized to produce sour pork as a functional food (China Patent No. ZL202110743486.8; Xu et al., 2024b). The favorable performance of this strain in food matrices suggests its potential as a probiotic. However, no systematic study has focused on *L. plantarum* DC2 lyoprotectants, gastrointestinal transient survival, or integrated *in vivo* intestinal morphology and microbiome evaluation.

Lyophilization is a dominant technique to prepare probiotic powder for storage stability (Guo X. et al., 2025; Aragon-Rojas et al., 2020). However, exposure to ultralow temperatures and dehydration causes severe decrease of survival rate, as intracellular ice crystal formation and lipid peroxidation disrupt bacterial membrane integrity (Guo X. et al., 2025; Aragon-Rojas et al., 2020; Noufeu et al., 2025). Formulation composition of lyoprotectant and preparation technology of probiotic powder are the decisive factors, and some previous studies have developed universal lyophilization protectant formulas and standard lyophilization procedures for *L. plantarum* (Guo X. et al., 2025; Aragon-Rojas et al., 2020). But most well-established universal lyoprotective systems are screened using common laboratory *L*. plantarum strains with robust intrinsic desiccation tolerance. Accordingly, their protective performance cannot be generalized to all conspecific strains. Distinct *L. plantarum* isolates exhibit considerable physiological heterogeneity, including divergent membrane fatty acid profiles, intracellular osmotic homeostasis and oxidative stress resistance (Sun et al., 2026; Cheng et al., 2022b). These physiological features directly govern strain-specific susceptibility to freeze-drying injury. Consistent with this notion, our preliminary trials verified that conventional generic protectant mixtures failed to retain high survival rate of *L. plantarum* DC2 during lyophilization, indicating that previously reported mature freeze-drying workflows cannot be directly adopted for *L. plantarum* DC2. Under standard lyophilization frameworks, poor survival rate of *L. plantarum* DC2 severely limits its development as a probiotic agent. Furthermore, most existing studies concerning *Lactiplantibacillus* lyophilization merely optimize post-drying viability as an isolated *in vitro* indicator (Guo X. et al., 2025; Aragon-Rojas et al., 2020; Noufeu et al., 2025; Sun et al., 2026; Cheng et al., 2022b). Few works integrate simulated gastrointestinal fluid tolerance tests to evaluate *in vivo* delivery efficiency, or validate intestinal-related effects through animal histological and microbiome analyses. Such one-dimensional experimental frameworks cannot construct a comprehensive evidence chain connecting lyoprotectant performance to host intestinal responses.

To further explore its storage stability and gastrointestinal regulatory value, the formula and process of lyophilized *L. plantarum* DC2 powder were optimized using single-factor design (SFD) and response surface methodology (RSM). Beyond lyophilization optimization alone, we supplemented simulated gastrointestinal digestion tests to quantify *L. plantarum* DC2 survival under digestive stress, and the effects of the probiotic powder on the murine gastrointestinal tract, including gut histopathology and microbiome composition, were also investigated. This work seeks to establish a scientific foundation for the development of *L. plantarum* DC2 as a novel probiotic supplement, providing systematic *in vitro* and *in vivo* strain-specific data that complements the current incomplete research framework of probiotic lyophilized preparations.

## Materials and methods

### Preparation and evaluation of lyophilized bacterial powder

The *L. plantarum* DC2 strain (CCTCC M 2019121), previously isolated and preserved in our laboratory (Xu et al., 2024b), was activated in de Man–Rogosa–Sharpe (MRS) liquid medium (cat. No. 02–293; Aoboxing Biotechnology, Beijing China) at 37 °C with shaking (180 rpm) until the OD600nm reached 1.2.

The bacterial cells were harvested via centrifugation (5,000 rpm/4,393 × g, 10 min, 4 °C), washed three times with sterile normal saline, and resuspended in sterile protectant solution at a 1:1 ratio (w/v, g/mL). The bacterial suspension was snap-frozen at −80 °C overnight prior to lyophilization (cat No. Scientz-10N, Ningbo Scientz Biotechnology Co. Ltd., China). The lyophilized bacterial powders were vacuum-sealed in aluminum foil bags (oxygen transmission rate < 0.1 cm^3^/m^2^/day) and stored at 20 °C, 4 °C, and −20 °C for 28 days. The lyophilized powder was rehydrated in sterile normal saline for subsequent analyses.

Viable bacterial counts were determined using the standard plate count method (Gitau et al., 2022; Kaymak Ertekin et al., 2024). Briefly, lyophilized bacterial powder was rehydrated to the original broth volume, vortexed thoroughly, and serially diluted (10^−6^-10^−9^). The dilutions were plated on MRS agar and incubated at 37 °C for 48 h. Colony-forming units (CFUs) were defined as the absolute number of viable colonies on the MRS plates, and the viable bacterial count per gram of lyophilized bacterial powder (CFU/g) was calculated as N1 × original broth volume/mass of lyophilized bacterial powder. The survival rate was calculated as follows: $Survival\;rate\;\left(\%\right)=\frac{N1}{N0}\times 100\%$, where *N0* is the initial viable cell count before lyophilization (CFU/mL), and *N1* is the postlyophilization viable cell count (CFU/mL).

### Protectant optimization via single-factor design (SFD) and response surface methodology (RSM)

The SFD and RSM experiments were performed according to previous methods (Wang et al., 2024; Xu et al., 2024c). Macromolecular protectants, including fat-free milk powder, maltodextrin, peptone and pectin, were tested at concentrations ranging from 20–160 g/L (w/v). Carbohydrate protectants, including saccharose, trehalose, xylooligosaccharide and oligofructose, were evaluated at concentrations ranging from 40–200 g/L. Polyols, including glycerine, mannitol, sorbitol and xylitol, were also evaluated at concentrations ranging from 40–200 g/L. Amino acid protectants, including L-glutamic acid, L-sodium glutamate and glycine, were tested at concentrations ranging from 20–100 g/L. The negative controls contained 0 g/L protectant in normal saline. Each protectant was tested in triplicate. On the basis of the SFD results, the optimal protectants from each category were selected as variables (four factors). A Box–Behnken design with three levels per factor was implemented (*n* = 29 experiments). The survival rate was the response variable and was calculated as previous description.

### Optimized preparation of lyophilized bacterial powder

The preparation of lyophilized bacterial powder was further optimized, including the ratio of bacterial cells to protectant, protectant pH, prefreezing temperature, prefreezing duration, and storage stability. The protectant pH was adjusted to 5.4, 5.8, 6.2 (as a control), 6.6, or 7.0 with 0.1 M HCl/NaOH and was precisely measured with a pH meter [cat No. ST3100, Ohaus Instruments (Changzhou) Co., Ltd., China]. Bacterial pellets (wet weight) were homogenized with protectant solution at mass–volume ratios of 1:0.5, 1:1 (as a control), 1:1.5, 1:2.0, and 1:2.5 (w/v) followed by 2 min of vortex mixing. The methods of prefreezing were also investigated, in which 10 mL of bacterial suspensions, contained in 50 mL polypropylene tubes, were rapidly frozen at −80 °C or gradually frozen at −20 °C or −40 °C for 12 h. Prefreezing duration optimization involved subjecting bacterial suspensions to −80 °C for 4, 8, or 12 h in a programmable ultralow-temperature freezer (cat No. MDF-86V338, Anhui Zhongke Duling Commercial Appliances Co., Ltd.) while maintaining ±2 °C temperature uniformity. The lyophilized bacterial powders were vacuum-sealed in aluminum foil bags (oxygen transmission rate < 0.1 cm^3^/m^3^/day), in which the moisture content was less than 3%. The bacterial powders were stored at 20 °C, 4 °C, or −20 °C for 28 d, with viable counts determined at 0, 2, 4, 7, 14, 21, and 28 d via triplicate plate counting on MRS agar.

### Animal experiments in ICR mice

Animal protocols were approved by the Institutional Animal Care and Use Committees of Huaihua University (2024A0110003). Sixty ICR mice (30♀/30♂, 7 weeks, 20 ± 2 g) were purchased from Changsha Tianqin Biotechnology Co., Ltd., and randomized into negative control (NC, 15♀/15♂) and lyophilized powder (LP, 15♀/15♂) groups. The mice were acclimated for 3 d under a 12 h light/12 h dark cycle with free access to food/water. At the end of the experiments, all the mice were deeply anesthetized by an intraperitoneal injection of 240 mg/kg avertin and then humanely euthanized by cervical dislocation to ensure death (the cessation of heartbeat and respiration, and the absence of a corneal reflex) prior to anatomical observation and sample collection.

For acute survival studies, the mice were subjected to intragastric (i.g.) administration of saline in the NC group or lyophilized powder solution in the LP group (2 × 10^8^ CFU/mouse in 10 μL/g). At 0, 30, 60, 90, 120 and 150 min postadministration, gastrointestinal contents, including stomach, duodenum, jejunum, ileum, caecum, and colorectum samples, excluding those 1 cm from junctions, were collected for viable cell count determination.

For subacute survival studies, the mice, including those in the NC and LP groups, received daily i.g. administration for 28 d. Body weight was monitored weekly. At termination, the intestinal tissues and contents of the small intestine (SI) and large intestine (LI) were collected. Gastrointestinal contents (excluding samples 1 cm from junctions) were used for viable *Lactobacillus* count determination and 16S rRNA sequencing. The tissue samples were processed for histopathological analysis.

### Histopathological evaluation

Histopathological evaluation was performed as described in our previous study (Xu et al., 2023, 2024a). Briefly, jejunum samples were collected and fixed, followed by dehydration, clearing, embedding, and sectioning (5 μm). After deparaffinization and rehydration, the sections were subjected to haematoxylin–eosin (H&E) staining. The sections were subsequently dehydrated, cleared and mounted on slides. After the sections were photographed, and the intestinal parameters were measured via a light microscope (BX53, Olympus) and Image-Pro Plus 6.0 software (Media Cybernetics). Five random fields per section were analyzed to determine the length of the intestinal villi and the depth of the intestinal villous crypts. All histological sections were evaluated under blinded conditions by three pathologists from Huaihua University. Five representative microscopic fields per section were randomly selected to measure intestinal villus length and crypt depth.

### Microbiome analysis

The contents (0.5 g) of the SI and LI were homogenized in sterile normal saline, serially diluted (10^−4^-10^−7^), and plated on MRS agar. The plates were incubated at 37 °C for 48 h, after which the number of colonies (30–300 CFU/plate) was counted.

Total DNA was extracted from the gut contents using an E.Z.N.A.^®^ Soil DNA Kit. The V3–V4 region of 16S rRNA was amplified with the primers 338F/806R and sequenced on an Illumina NextSeq2000 platform. The sequences were processed via fastp, FLASH, and UPARSE (97% identity). Taxonomic assignment was performed with the RDP Classifier (≥70% confidence). The functional profile was predicted via PICRUSt2. Bioinformatics analysis was conducted on the Majorbio Cloud platform (https://cloud.majorbio.com; Han et al., 2024).

### Statistical analysis

The data were analyzed using SPSS 19.0. Normality was tested with the Shapiro–Wilk test. Parametric data were compared via one-way/two-way ANOVA with the Bonferroni *post-hoc* test. Non-parametric data were analyzed by the Kruskal–Wallis test. The results are presented as the means ± SDs, with significance set at *p*<*0.05* (^*^ or different lowercase letters) or *p* < 0.01 (^**^ or different capital letters). No marking or the same letters indicate no significant difference.

For gut microbiota differential abundance analysis and PICRUSt2 functional prediction, all multi-group comparisons were automatically processed with FDR multiple testing correction embedded in the bioinformatic pipeline to control false-positive results. Only taxa and functional pathways with adjusted *p* < 0.05 (^*^) or *p* < 0.01 (^**^) after FDR correction were regarded as significantly differential. Simple pairwise comparisons without additional manual FDR correction were only used as auxiliary reference data and not adopted to screen core differential biomarkers.

## Results

### The protectant compositions of the lyophilized bacterial powder

The SFD results revealed optimal protectants across the four categories. Compared with normal saline, macromolecular protectants significantly improved the survival rate (*p* < 0.05). Fat-free milk powder increased the survival rate in a concentration-dependent manner (20–160 g/L), with the maximum survival rate of 72.37% observed at 160 g/L. The survival rate showed a parabolic response to maltodextrin (a peak of 46.21% at 80 g/L). However, peptone and pectin failed to form stable solutions above 100 g/L. At concentrations lower than 100 g/L, peptone increased the survival rate in a concentration-dependent manner (a peak of 48.36% at 100 g/L), whereas the survival rate showed a parabolic response to pectin (a peak of 63.91% at 40 g/L; Figure 1A).

**Figure 1:**
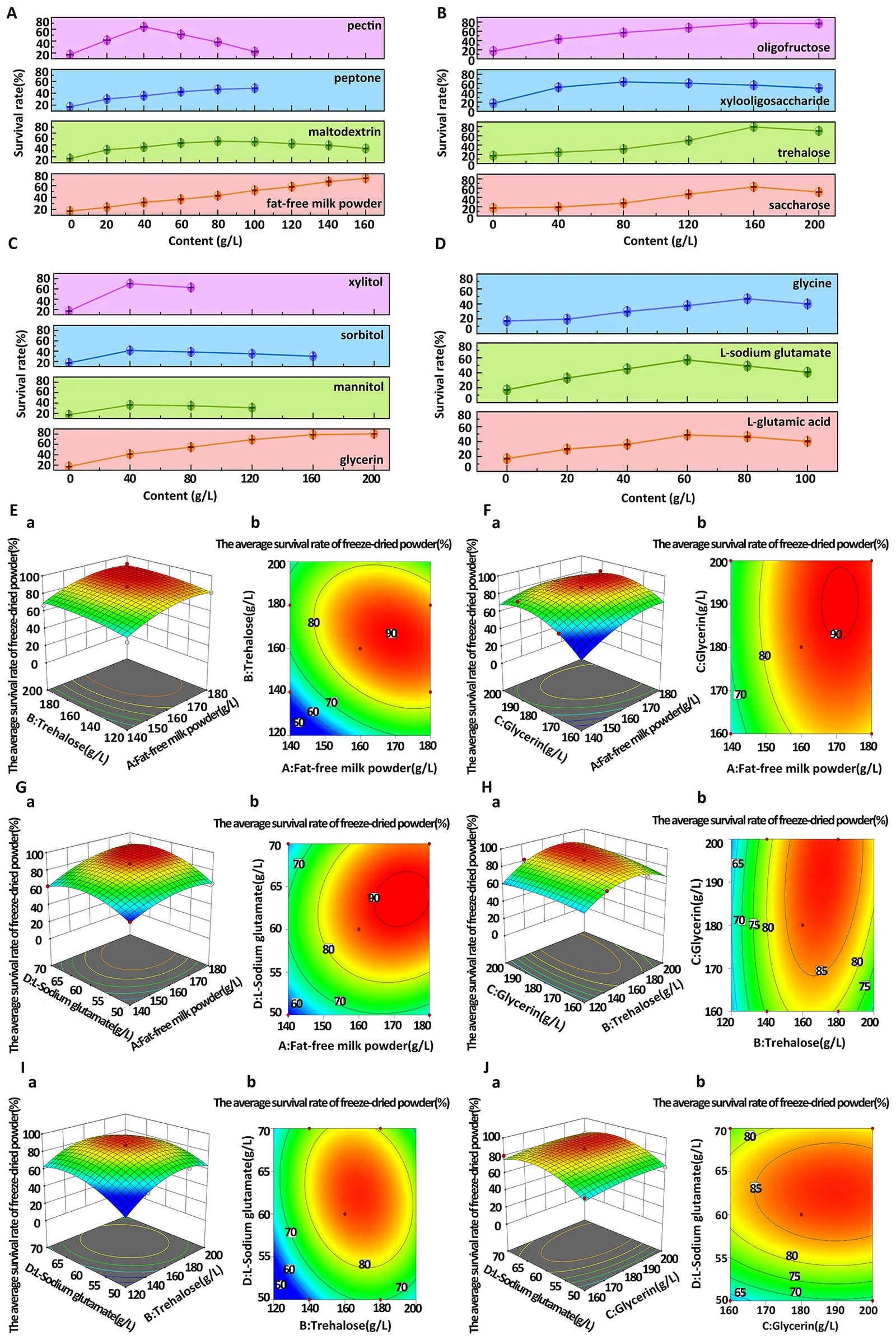
Selection and optimization of protectants for lyophilized bacterial powder based on SFD and RSM. **(A)** Macromolecular protectants, including fat-free milk powder, maltodextrin, peptone and pectin. **(B)** Carbohydrate protectants, including saccharose, trehalose, xylooligosaccharide and oligofructose. **(C)** Polyol protectants, including glycerine, mannitol, sorbitol and xylitol. **(D)** Amino acid protectants, including L-glutamic acid, L-sodium glutamate and glycine. **(Ea, Eb)** 3D response surfaces and 2D contour plots of the correlation between fat-free milk powder **(A)** and trehalose **(B)**. **(Fa, Fb)** 3D response surfaces and 2D contour plots of the correlation between fat-free milk powder **(A)** and glycerine **(C)**. **(Ga, Gb)** 3D response surfaces and 2D contour plots of the correlation between fat-free milk powder **(A)** and L-sodium glutamate **(D)**. **(Ha, Hb)** 3D response surfaces and 2D contour plots of the correlation between trehalose **(B)** and glycerine **(C)**. **(Ia, Ib)** 3D response surfaces and 2D contour plots of the correlation between trehalose **(B)** and L-sodium glutamate **(D)**. **(Ja, Jb)** 3D response surfaces and 2D contour plots of the correlation between glycerine **(C)** and L–sodium glutamate **(D)**.

Among the carbohydrates, trehalose had the strongest effect on the survival rate (peak of 79.46% at 160 g/L), followed by oligofructose (peak of 77.23% at 160 g/L), xylooligosaccharide (peak of 63.44% at 80 g/L) and saccharose (peak of 62.73% at 160 g/L), which provided moderate protection at optimal concentrations (Figure 1B). Glycerol was the most effective polyol (peak of 80.36% at 160 g/L), whereas mannitol, sorbitol, and xylitol were less effective at concentrations above 40 g/L (Figure 1C). Among the amino acids, L-sodium glutamate provided the best protection (peak of 57.29% at 60 g/L), outperforming L-glutamic acid and glycine (Figure 1D). Therefore, fat-free milk powder, trehalose, glycerine and L-sodium glutamate were optimal protectants for maintaining the survival rates of lyophilized bacterial powder.

### The optimum formula of the lyophilized bacterial powder

On the basis of the SFD results, four factors (A: fat-free milk powder, B: trehalose, C: glycerine, D: L-sodium glutamate) were selected for the Box–Behnken RSM. The quadratic regression model (Equation 1) was highly significant (*F* = 19.39, *p* < 0.0001), with acceptable predictive accuracy (LOF= 154.40 (*p* = 0.1962 > 0.05), *R*^2^ = 0.9510, and adjusted *R*^2^ = 0.9019). The factor significance ranking was A > D > B > C, with quadratic terms dominating interactions.


$$\begin{array}{l} Y=87.4+8.73A+3.56B+2.95C+6.49D-2.07BD-8.13A^{2}\\ \;-3.92B^{2}-3.10C^{2}-12.24D^{2} \end{array}$$ (1)

The optimized formulations were composed of 180.00 g/L fat-free milk powder, 163.14 g/L trehalose, 182.10 g/L glycerine, and 62.27 g/L L-sodium glutamate. The predicted survival rate was 89.92%. Experimental validation yielded an 88.98% survival rate, demonstrating the reliability of the model. This represented a 5.19-fold improvement over the saline control. Three-dimensional response surfaces revealed concentration optima for each factor pair, with fat-free milk powder exerting the strongest influence (Figures 1E–J).

### Storage stability of the lyophilized bacterial powder

The pH of the protectant significantly affected bacterial survival during lyophilization. The survival rate increased from 72.30% at pH 5.4 to a peak of 89.91% at pH 6.6, followed by a decrease to 75.20% at pH 7.0. The optimal pH was determined to be 6.6 (Figure 2A). The bacterial survival rate exhibited a parabolic response to the protectant ratio. The maximum survival rate (94.24%) was achieved at a 1:1.5 (w/v) ratio. Low ratios ( ≤ 1:1) provided insufficient protection, whereas high ratios (≥1:2) impaired cell permeability (Figure 2B). Prefreezing at −80 °C for 12 h yielded the highest survival rate (93.45%), which was significantly better than that observed with prefreezing at −20 °C (68.10%) or −40 °C (79.30%). Rapid freezing at −80 °C minimized cellular damage (Figure 2C). Prefreezing for 4 h at −80 °C resulted in a survival rate of 92.10%, which was comparable to that observed with longer durations. Extending prefreezing beyond 4 h did not increase the survival rate but increased energy consumption, leading to the selection of 4 h as the optimal duration (Figure 2D). The storage temperature profoundly affected the survival rate. At 20 °C, the viable bacterial cell counts decreased from 1.65 × 10^12^ to 0.15 × 10^12^ CFU/g within 28 d (9.09% survival rate), with 70% loss occurring during the first week. Storage at 4 °C provided moderate stability (0.85 × 10^12^ CFU/g, 51.52% survival rate), whereas storage at −20 °C maintained the highest stability (1.45 × 10^12^ CFU/g, 87.88% survival rate) after 28 d (Figure 2E). These results indicated that −20 °C was the optimal storage temperature for preserving the bacterial survival rate.

**Figure 2:**
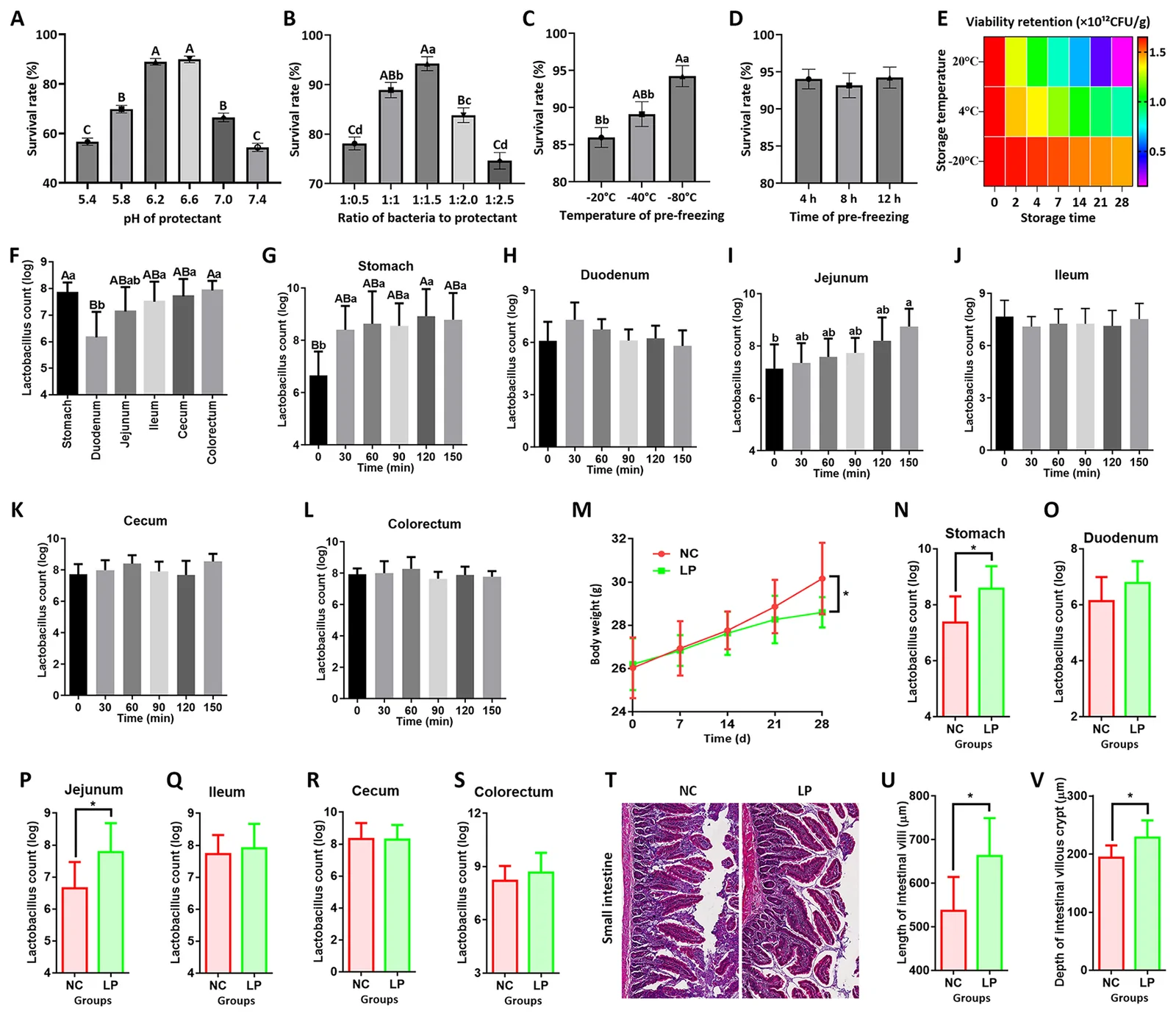
Optimization of the preparation processes for lyophilized bacterial powder and exploration of its effects on mice. **(A–D)** Effects of the pH of the protectant, the ratio of bacteria to protectant, the prefreezing temperature and the duration of pre-freezing on the survival rate of bacteria. **(E)** Effects of storage conditions (time and temperature) on survival rate retention. **(F)** *Lactobacillus* contents in each section of the digestive tract, including the stomach, duodenum, jejunum, ileum and colorectum. **(G–L)** *Lactobacillus* contents in the stomach, duodenum, jejunum, ileum and colorectum at 0, 30, 60, 90, 120 and 150 min after a single *Lactobacillus* administration. **(M)** Increases in body weight after continuous *Lactobacillus* administration for 28 d. **(N–S)** *Lactobacillus* contents in the stomach, duodenum, jejunum, ileum and colorectum after continuous *Lactobacillus* administration for 28 d. **(T)** Tissue sections and H&E staining of the jejunum. **(U)** Analysis of intestinal villus length. **(V)** Analysis of villous crypt depth. Parametric data were compared via one-way ANOVA with the Bonferroni *post-hoc* test or independent-samples *t*-test. Different lowercase letters or * (*p* < 0.05) and different capital letters (*p* < 0.01) or indicate significant differences. No marking or the same letters indicate no significant difference.

### Effects of lyophilized bacterial powder on gastrointestinal *Lactobacillus* strains

In the gastrointestinal tract of the mice without any interruption, the number of *Lactobacillus* strains in the duodenum was significantly lower than that in the stomach, jejunum, ileum, caecum, and colon (Figure 2F). Thirty minutes after the intragastric administration of *L. plantarum* DC2, the number of *Lactobacillus* strains in the stomach was significantly greater than that before administration, and there was no significant difference in the number of *Lactobacillus* strains in the stomach between 30 and 150 min after administration (Figure 2G). Intragastric administration of *L. plantarum* DC2 did not significantly alter the number of *Lactobacillus* strains in the duodenum (Figure 2H). After the intragastric administration of *L. plantarum* DC2, the number of *Lactobacillus* strains in the jejunum increased significantly over time (Figure 2I). Intragastric administration of *L. plantarum* DC2 did not significantly affect the numbers of *Lactobacillus* strains in the ileum, caecum, or colon (Figures 2J–L). Continuous intragastric administration of *L. plantarum* DC2 for 28 d slowed the weight gain of the mice in the LP group, which was significantly lower than that in the NC group (Figure 2M). The number of *Lactobacillus* strains in the stomach increased significantly (Figure 2N). The number of *Lactobacillus* strains in the duodenum also increased but not significantly (Figure 2O). The number of *Lactobacillus* strains in the jejunum increased significantly (Figure 2P). The numbers of *Lactobacillus* strains in the ileum, caecum, and colon increased but not significantly (Figures 2Q–S). The continuous intragastric administration of *L. plantarum* DC2 for 28 d significantly increased the length and complexity of the villi and the depth of the crypts in the intestine (Figures 2T–V).

### Effects of lyophilized bacterial powder on intestinal microbial composition and abundance

The Chao1 index values indicated that the microbial diversity in the NC group was generally greater than that in the LP group (*p* = 0.01221; Figure 3A), and the microbial diversity in the large intestine (LI) was also greater than that in the small intestine (SI) (*p* = 0.04503; Figure 3B). The microbial compositions in the SI and LI of the NC and LP groups were analyzed with a Venn diagram. The NC group contained 1140 OTUs, and the LP group contained 644 OTUs. A total of 473 OTUs were shared between the NC and LP groups (Figure 3C). There were 1,128 OTUs in the LI and 681 OTUs in the SI, of which 498 OTUs were shared between the LI and the SI (Figure 3D). Within the NC group, there were 945 OTUs in the large intestine of the NC group (NCLI) and 626 OTUs in the small intestine of the NC group (NCSI), of which 431 OTUs were shared between the NCLI and the NCSI. Within the LP group, there were 533 OTUs in the large intestine of the LP group (LPLI) and 322 OTUs in the small intestine of the LP group (LPSI), of which 211 OTUs were shared between the LPLI and the LPSI. There were 399 unique OTUs in the NCLI, 133 unique OTUs in the NCSI, 141 unique OTUs in the LPLI, and 15 unique OTUs in the LPSI (Figure 3E). The results suggested that the gut microbiota was altered after subacute administration of *L. plantarum* DC2, most significantly in the SI.

**Figure 3:**
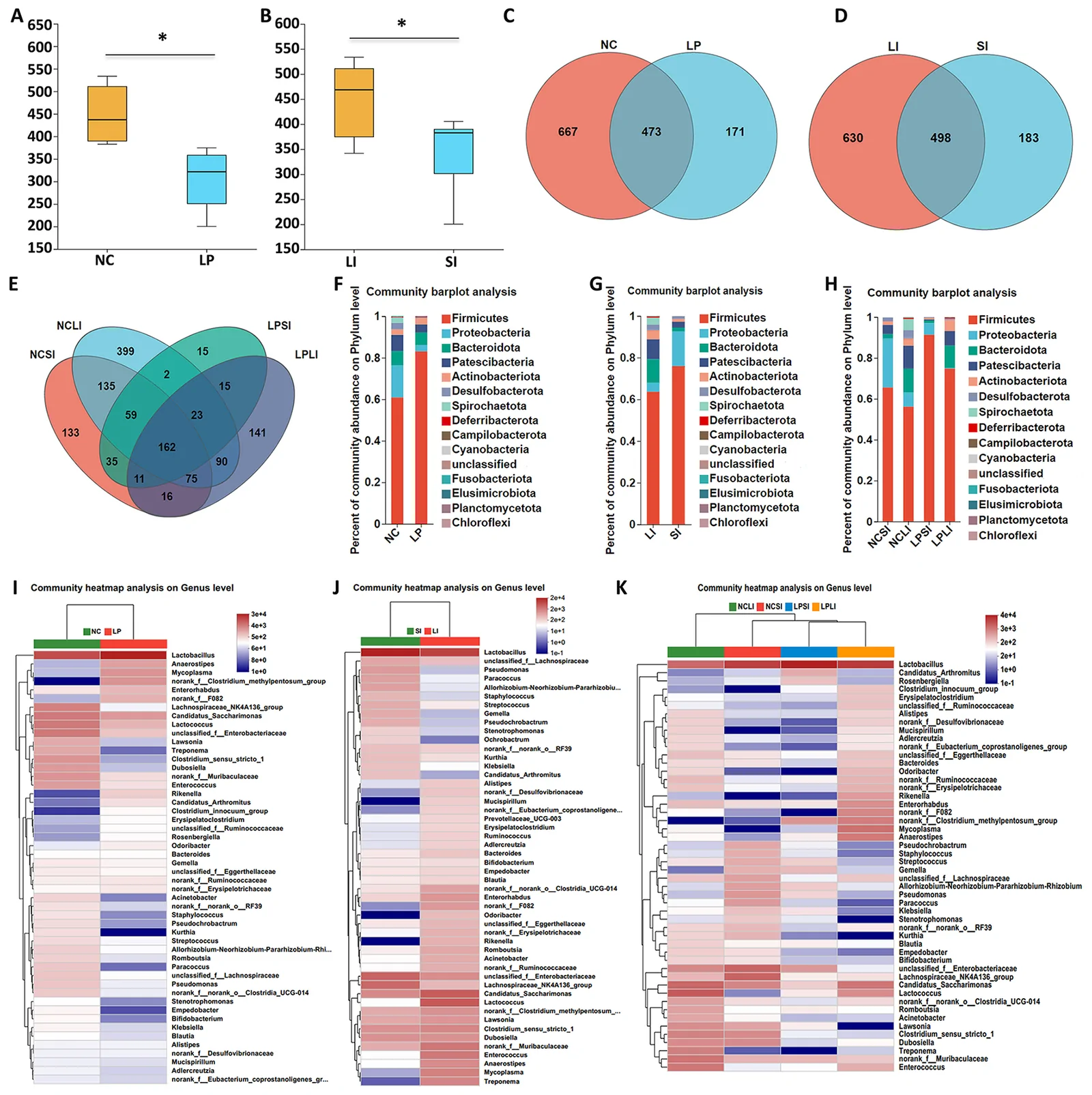
Group analysis of intestinal microbiota after continuous *Lactobacillus* administration for 28 d. **(A)** Alpha diversity comparison between the NC and LP groups. **(B)** Alpha diversity comparison between the LI and the SI. **(C)** Venn diagrams of the OTUs in the NC and LP groups. **(D)** Venn diagrams of the OTUs in the LI and the SI. **(E)** Venn diagrams of the OTUs in the NCSI, NCLI, LPSI and LPLI. **(F)** Community abundance of the intestinal microbiota at the phylum level in the NC and LP groups. **(G)** Community abundance of the intestinal microbiota at the phylum level in the LI and the SI. **(H)** Community abundance of the intestinal microbiota at the phylum level in the NCSI, NCLI, LPSI and LPLI. **(I)** Community abundance of the intestinal microbiota at the genus level in the NC and LP groups. **(J)** Community abundance of the intestinal microbiota at the genus level in the LI and the SI. **(K)** Community abundance of the intestinal microbiota at the genus level in the NCSI, NCLI, LPSI and LPLI. Differential taxa were identified based on FDR-adjusted *P*-values from multi-group comparisons. Parametric data were compared via independent-samples *t*-test. *(*p* < 0.05) indicate significant differences. No marking or the same letters indicate no significant difference.

Compared with those in the NC group, the percent community abundance at the phylum level in the LP group significantly changed, for example, the abundances of *Firmicutes* and *Actinobacteriota* significantly increased, and the abundances of *Proteobacteria, Patescibacteria, Desulfobaceterota* and *Spirochaetota* significantly decreased (Figure 3F). There were also significant differences between the LI and the SI, such as in the abundances of *Firmicutes, Proteobacteria*, and *Bacteroidota* (Figure 3G). Within the NC group, the abundances of *Firmicutes* and *Proteobacteria* in the NCLI were significantly lower than those in the NCSI, and the abundances of *Bacteroidota, Actinobacteria, Patescibacteria* and *Spirochaetota* in the NCLI were significantly greater than those in the NCSI. After subacute administration of *L. plantarum* DC2 within the LP group, these trends did not change, but the abundance of *Spirochaetota* significantly decreased (Figure 3H).

The community abundance at the genus level in the LP group significantly differed from that in the NC group, suggesting that the abundance of gut microorganisms changed significantly after subacute administration of *L. plantarum* DC2. Compared with those in the NC group, the abundances of some microorganisms, including *Lactobacillus, Anaerostipes*, and *Enterorhabdus*, increased. However, the abundances of some other microorganisms, including *Treponema, Kurthia*, and *Empedobacterium*, decreased (Figure 3I). Compared with those in the SI, the abundances of some microorganisms, including *Mucispirillum, Odoribacter*, and *Rikenella*, increased. However, the abundances of several other microorganisms, including *Pseudomonas, Gemella*, and *Ochrobactrum*, decreased (Figure 3J). Within the NC group, the abundances of *Mucispillum, Treponema* and *Lactococcus* in the NCLI were significantly greater than those in the NCSI, and the abundances of *Paracoccus, Gemella* and *Pseudomonas* in the NCLI were significantly lower than those in the NCSI. Within the LP group, these trends for some microorganisms were reversed (Figure 3K).

### Effects of lyophilized bacterial powder on the intestinal indices

To explore the β diversity of the intestinal microbial communities between the LI and the SI or between the NC and LP groups, principal coordinate analysis (PCoA) was performed. The distribution of microbial community points in the LP group was distinct from that in the NC group, and the PC2 value in the LP group was lower than that in the NC group (Figure 4A). The distribution of microbial community points in the LI was separate from that in the SI, and the PC2 value in the LI was lower than that in the SI (Figure 4B).

**Figure 4:**
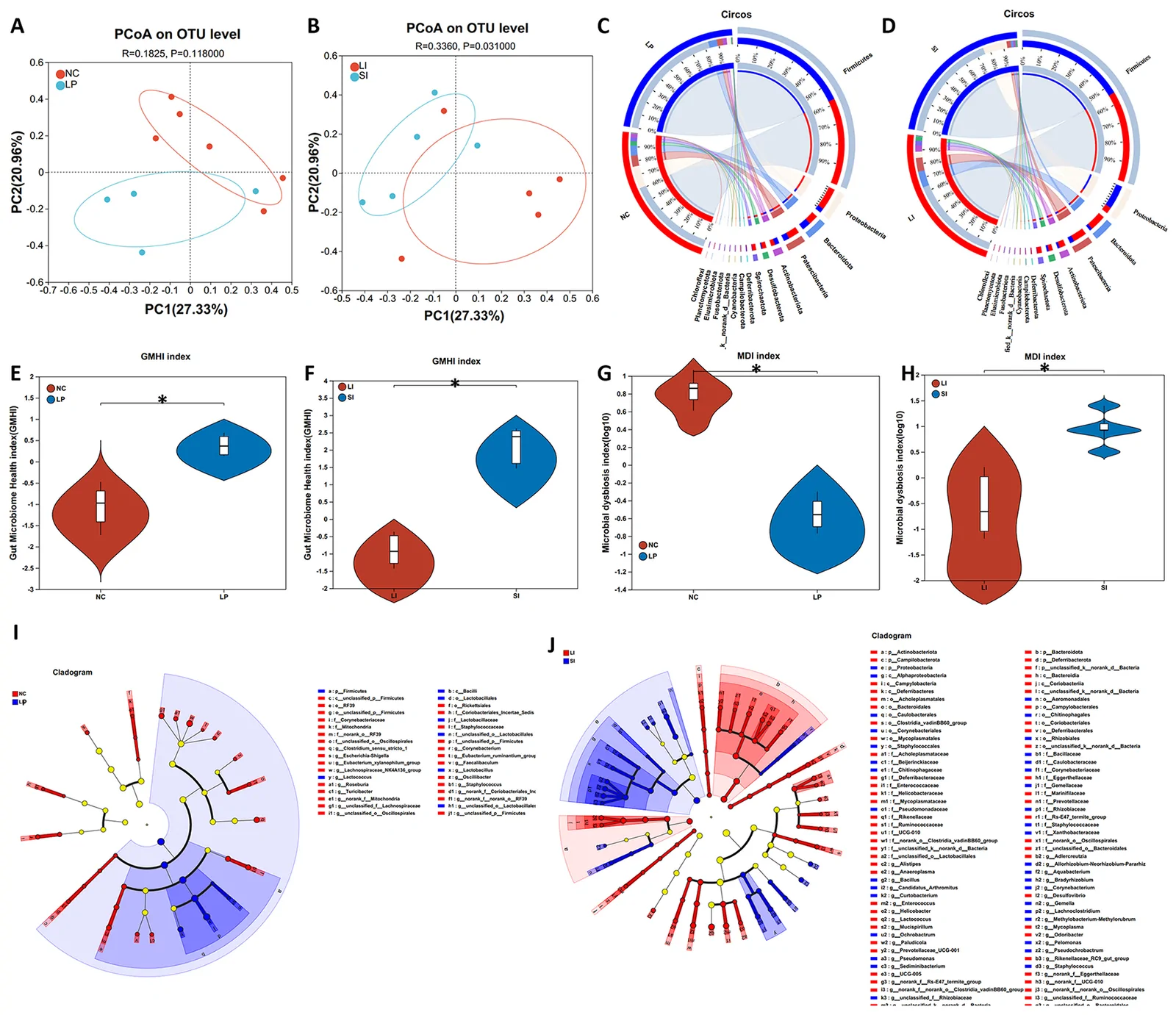
Analysis of intestinal health status after continuous *Lactobacillus* administration for 28 d. **(A)** PCoA map of the intestinal microbiota in the NC and LP groups. **(B)** PCoA map of the intestinal microbiota in the LI and the SI. **(C)** Circos sample and species map of the intestinal microbiota in the NC and LP groups. **(D)** Circos sample and species map of the intestinal microbiota in the LI and the SI. **(E)** GMHI index map of the intestinal microbiota in the NC and LP groups. **(F)** GMHI index map of the intestinal microbiota in the LI and the SI. **(G)** MDI index map of the intestinal microbiota in the NC and LP groups. **(H)** MDI index map of the intestinal microbiota in the LI and the SI. **(I)** LEfSe branching plot of the intestinal microbiota in the NC and LP groups. **(J)** LEfSe branching plot of the intestinal microbiota in the LI and the SI. Differential taxa were identified based on FDR-adjusted *P*-values from multi-group comparisons. Parametric data were compared via independent-samples *t*-test. *(*p* < 0.05) indicate significant differences. No marking or the same letters indicate no significant difference.

The bacterial community compositions and their interrelations between the NC and LP groups are displayed as Circos plots, where connecting lines represent the associations between different bacteria. The thicker the line is, the stronger the association. Bacteria such as *Proteobacteria* (red), *Firmicutes* (blue), and *Bacteroidota* (green) had the greatest connections with the thickest lines, indicating that these three bacterial phyla were strongly interconnected. In contrast, associations between other bacterial phyla were relatively weak. The intestinal microbiota diversity in the LP group was greater than that in the NC group, suggesting that subacute administration of *L. plantarum* DC2 resulted in the inhibition of the growth of non-beneficial microorganisms (Figure 4C). The intestinal microbiota diversity in the SI was simpler than that in the LI, and subacute administration of *L. plantarum* DC2 had more significant effects on the SI microbiota (Figure 4D).

The gut microbiome health index (GMHI) is a robust index for assessing health status and uses species-level taxonomic features of gut microbiome samples to determine the extent of disease. This index is used primarily to determine the likelihood of illness independent of clinical diagnosis by comparing the relative abundances of microbial taxa associated with both good and poor health conditions (Yin et al., 2024; Zhao et al., 2025; Gupta et al., 2020). Compared with alpha diversity metrics, the GMHI exhibits superior discriminatory power between healthy and unhealthy groups, showing strong reproducibility in stratifying individuals into these categories. It is often considered a marker of gut health and dysbiosis. On the basis of this definition, the GMHI of the LP group indicated a healthier status than that of the NC group (*p* = 0.01421; Figure 4E). The GMHI in the SI indicated a healthier status than that in the LI (*p* = 0.01219; Figure 4F).

The gut dysbiosis index (MDI) is a measure of the degree of microbial ecological dysbiosis. The higher the index is, the greater the degree of microbial dysregulation (Yin et al., 2024; Zhao et al., 2025; Gupta et al., 2020). On the basis of this definition, the MDI in the LP group was lower than that in the NC group (*p* = 0.01421; Figure 4G). The MDI in the SI was greater than that in the LI (*p* = 0.01219; Figure 4H).

Linear discriminant analysis effect size (LEfSe) was used to identify differentially abundant species within microbial communities between the NC and LP groups (Figure 4I), and between the LI and the SI (Figure 4J), with different colors representing different stages of decomposition. The taxonomic hierarchy from the phylum to the genus level, with node sizes indicating the average relative abundance of the taxonomic units, is displayed as a cladogram. Both hollow and colored nodes significantly differed in terms of their intergroup variability and relatively high abundance.

### Predicted functional profiles of the gut microbiota

Functional profiles of the gut microbiota were predicted using PICRUSt2 based on 16S rRNA gene sequencing, and the predicted abundances of enzymes, KO terms, modules, and KEGG pathways at different hierarchical levels are visualized as heatmaps (Figure 5). At the enzyme level (Figure 5A), several key enzymes involved in carbohydrate and amino acid metabolism showed differential abundance between the NC and LP groups. Notably, the LP group exhibited relatively higher predicted abundance of enzymes related to core metabolic processes, while some enzymes associated with other functions were decreased compared with the NC group. At the KO (orthology) level (Figure 5B), functional units involved in central metabolism, including carbohydrate and amino acid turnover, were among the most abundant across all groups. Several KO terms linked to these pathways were enriched in the LP group, whereas some terms related to environmental information processing and other functions were reduced.

**Figure 5:**
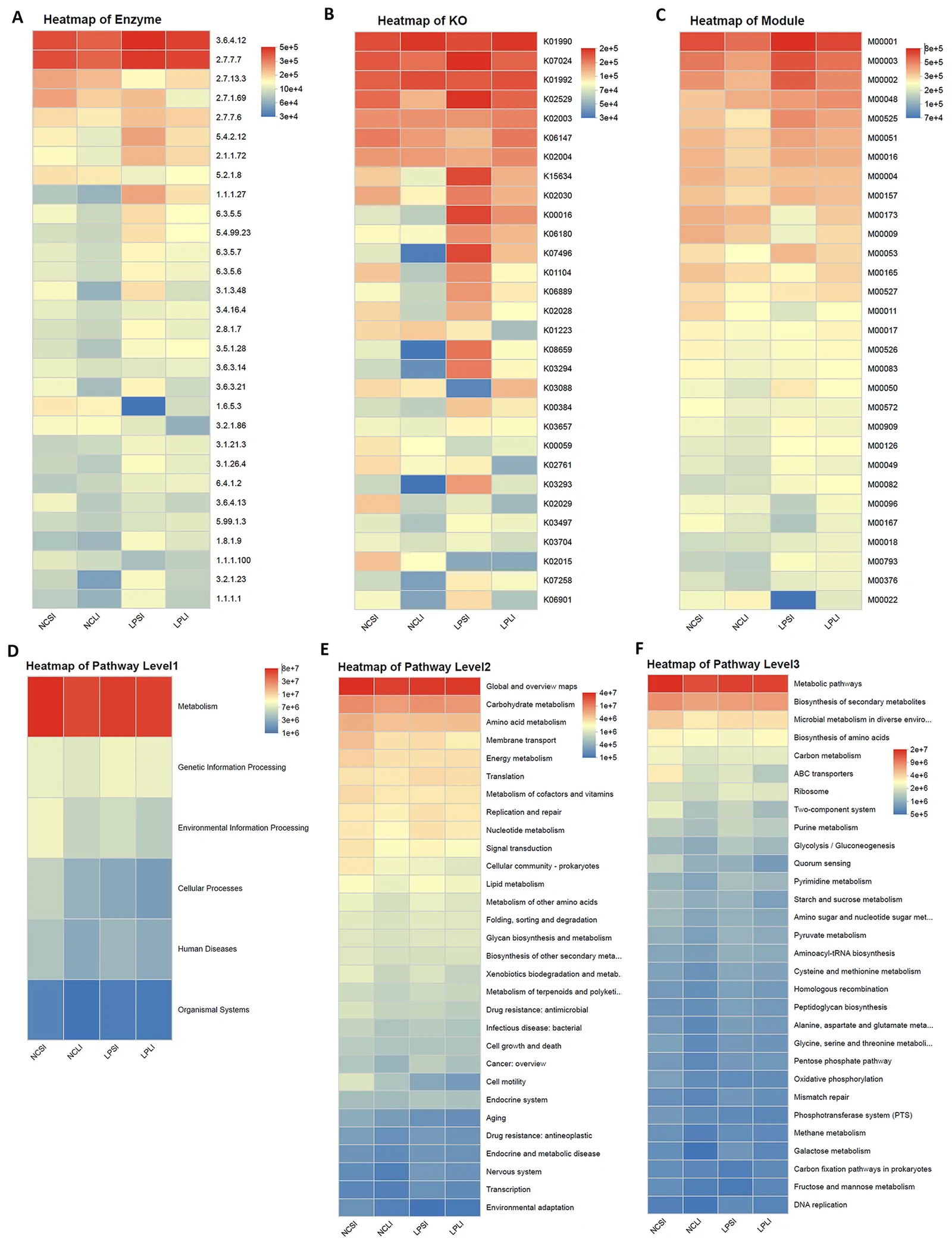
Heatmaps of PICRUSt2-predicted functional profiles of the gut microbiota at different hierarchical levels. **(A–C)** Heatmap of predicted enzyme, KO (orthology) and module abundances, respectively. **(D–F)** Heatmap of predicted KEGG pathway abundances at the level 1, level 2 and level 3. Rows represent functional categories/units, and columns represent samples from different experimental groups (NCSI, NCLI, LPSI, LPLI). Differential taxa were identified based on FDR-adjusted *P*-values from multi-group comparisons. Color intensity indicates the relative abundance of each function, with red representing higher abundance and blue representing lower abundance. All functional profiles were predicted using PICRUSt2 based on 16S rRNA gene sequencing data. Note that these are bioinformatic predictions and require further experimental validation.

At the module level (Figure 5C), modules associated with metabolic pathways, particularly those involved in amino acid biosynthesis and carbohydrate metabolism, were generally dominant. The LP group showed higher predicted abundance of core metabolic modules, while modules related to other processes were less abundant compared with the NC group. At KEGG Pathway Level 1 (Figure 5D), Metabolism was the dominant functional category, showing consistently high abundance in all groups. In contrast, Organismal Systems and Human Diseases exhibited the lowest predicted abundances. The LP group showed a slight increase in the relative abundance of Metabolism pathways compared with the NC group. At KEGG Pathway Level 2 (Figure 5E), carbohydrate metabolism, amino acid metabolism, membrane transport, energy metabolism, and translation were the most abundant functional categories across all groups. Compared with the NC group, the LP group showed a slight increase in carbohydrate and amino acid metabolism, while pathways related to environmental adaptation and infectious diseases were relatively decreased. At KEGG Pathway Level 3 (Figure 5F), specific pathways including biosynthesis of secondary metabolites, microbial metabolism in diverse environments, biosynthesis of amino acids, carbon metabolism, and ABC transporters were the most abundant. Consistent with the higher-level classifications, the LP group showed increased predicted abundance of core metabolic pathways, such as carbon metabolism and amino acid biosynthesis, while pathways related to DNA replication and environmental adaptation were relatively decreased. It should be noted that these functional profiles are based on bioinformatic predictions only and have not been experimentally validated. Future studies are required to confirm the biological relevance of these predicted changes.

## Discussion

Exploration of various protectants revealed that specific types significantly improved the survival rate of *L. plantarum* DC2 during lyophilization and preservation. This protective effect may be attributed to two key mechanisms. First, these protectants were absorbed to the bacterial cell surfaces and formed a protective layer that mitigated mechanical damage caused by ice crystal nucleation and growth during freezing, cell dehydration of sublimation, and preservation. Second, these protectants provide a stable microenvironment for dormant bacteria to enter a dormant state with minimal metabolic activity by maintaining the native conformation of cell membrane lipids and proteins, thereby preventing irreversible denaturation (Gao et al., 2022).

However, some single-component protectants exhibited reduced efficacy at relatively high concentrations. These phenomena were potentially driven by hyperosmotic stress, as elevated solute concentrations disrupted the osmotic balance across the bacterial cell membrane, leading to excessive water efflux and cell shrinkage. Additionally, nutrient saturation at high protectant concentrations increases the unnecessary metabolic burden on dormant bacterial cells, further compromising their survival rate (Gao et al., 2022).

A critical finding was that composite protectants outperformed their single-component counterparts in ensuring comprehensive protection during lyophilization. This superiority stems from synergistic interactions between components. For example, glycerine primarily protected cell membranes by insertion into lipid bilayers, whereas fat-free milk powder and trehalose stabilized intracellular proteins via hydrogen bonding and inhibited ice recrystallization. These synergistic effects were not achieved with any single protectant alone (Chang et al., 2021).

In the preparation of the lyophilized bacterial powder, the ratio of the bacterial slurry to the protectants emerged as a key determinant of postlyophilization survival rate. The two scenarios led to a significant decrease in the final survival rate. When the bacterial density was excessively high or the protectant content was insufficient, a relatively large proportion of the bacterial cells were not fully coated by the protectant layer, resulting in direct exposure to freezing-induced ice crystal damage, and sublimation-induced dehydration during lyophilization resulted in an increase in the mortality rate during our preliminary experiments (Cheng et al., 2022a). When the bacterial density was too low or the protectant content was excessive, the hyperosmotic environment severely disrupted cell membrane permeability, as evidenced by increased leakage of intracellular nucleic acids and reduced membrane potential, which resulted in a reduction in the cell survival rate (Cheng et al., 2022a). Therefore, optimizing the ratio of slurry to protectant was not merely a quantitative adjustment, but also a crucial step to ensure uniform protectant coverage while avoiding osmotic stress and directly supporting the need for the ratio optimization conducted in this study.

Furthermore, the pre-freezing temperature (−80 °C) played a critical role in maintaining bacterial survival rate through the determination of two interrelated factors, namely, the freezing rate and ice crystal size. During lyophilization, an appropriate prefreezing temperature promoted a moderate freezing rate, which restricted the growth of ice crystals and minimized mechanical damage to bacterial structures, such as cell membranes and intracellular organelles. Conversely, inappropriate prefreezing temperatures (e.g., −20 °C or −40 °C) caused gradual freezing, which can induce intracellular ice crystal formation, which is another major cause of cell death (Wang et al., 2025; Yang et al., 2022). Thus, the selection of an optimal prefreezing temperature was not arbitrary but was an essential strategy to balance the ice crystal size and freezing rate, thereby increasing the survival rate of the lyophilized bacterial powder.

With respect to storage conditions, different temperatures significantly affected the bacterial survival rate and storage stability. At low temperatures (4 °C and −20 °C), the activity of metabolic enzymes in bacterial cells markedly reduced, maintaining the bacterial cells in a low physiological or dormant state. This dormancy minimizes the consumption of intracellular reserves and reduces the accumulation of reactive oxygen species (ROS), which are the key factors contributing to cell senescence. Conversely, at elevated temperatures (>20 °C), bacterial cells remained metabolically active, but the lack of sufficient nutrients, water and other appropriate conditions, and the accumulation of toxic metabolic byproducts accelerated bacterial senescence and even death (Bottino et al., 2024). Therefore, choosing appropriate storage temperatures is vital for preserving the storage stability and high survival rate of lyophilized bacterial powder.

*Lactobacillus* species exhibit distinct survival and colonization characteristics across different segments of the mouse gastrointestinal tract. Acute intake of *L. plantarum* DC2 significantly increased the abundance of *Lactobacillus* in the stomach and SI sections, but this increase was ecologically unstable. Importantly, these changes in *Lactobacillus* abundance did not result from modifications to the indigenous intestinal bacterial community structure, but rather reflected the survival and transient residence of exogenously administered *L. plantarum* DC2 from the lyophilized powder (Jiang et al., 2025). This transient presence was a direct consequence of normal gastrointestinal physiology. As the gastrointestinal tract undergoes peristalsis and gastric/intestinal emptying, a subset of ingested *L. plantarum* DC2 cells transiently resided in different gastrointestinal segments at 0–150 min before being further transported to the distal gut. Although transient, this survival was biologically meaningful, from which it was confirmed that *L. plantarum* DC2 had withstood the harsh environment of the upper gastrointestinal tract to reach the distal gut, a prerequisite for exerting probiotic effects (Jiang et al., 2025).

To increase the probiotic potential of *L. plantarum* DC2, the period of intervention was extended to subacute intake. This regimen led to three key outcomes. First, the abundance of *Lactobacillus* in the entire gastrointestinal tract was significantly increased, effectively reducing the diversity and abundance of conditional pathogens or non-beneficial bacteria such as *Spirilla* (Qin et al., 2022). Second, subacute intake of lyophilized bacterial powder promoted histological adjustments, including the remodeling of intestinal structure, to increase villus length and complexity and crypt depth, and to maintain mucosal integrity. These structural changes directly support enhanced barrier function (Qin et al., 2022). Third, supplementation with *L. plantarum* DC2 reshaped gut microecology, potentially optimized nutrient absorption, and enabled competitive exclusion against pathogenic microbes (Jiang et al., 2025). Additionally, *L. plantarum* DC2 intervention resulted in a slowdown in weight gain in mice (Shirouchi et al., 2016). Notably, different effects of *L. plantarum* DC2 were observed between the SI and the LI. This discrepancy was likely associated with segment-specific gastrointestinal conditions. The SI had a higher pH (6.5–7.5) and faster transit time. Conversely, the LI had a lower pH (5.0–6.0) and a richer microbial community. These potential mechanisms contributed to the survival, metabolism and colonization capacity of *L. plantarum* DC2 (Van Hul et al., 2024).

Two potential mechanisms may account for the observed intestinal phenotypic alterations triggered by *L. plantarum* DC2 supplementation. First, *L. plantarum* DC2 may produce antimicrobial substances such as organic acid and bacteriocins. Organic acids can lower the intestinal pH to 4.5–5.5, creating an acidic environment that inhibits the growth of many pH-sensitive pathogens (Du et al., 2025). Moreover, bacteriocins exert targeted antibacterial activity by disrupting the cell membranes of pathogenic bacteria, leading to their lysis (Du et al., 2025). Second, *L. plantarum* DC2 may interact with intestinal immune cells to shift the cytokine profile toward an antiinflammatory state, specifically by upregulating the production of antiinflammatory cytokines and downregulating the production of proinflammatory cytokines. This immune modulation reduces excessive intestinal inflammation and preserves mucosal homeostasis (Guo J. et al., 2025). Notably, these mechanistic hypotheses are inferred based on published research concerning *Lactobacillus* strains. Further targeted experimental verification is required to clarify the causal relationships between DC2 supplementation and the observed intestinal phenotypic alterations.

This study has several notable limitations that should be acknowledged for a comprehensive interpretation of the present results. First, viable bacterial counts using MRS agar reflect only the total abundance of *Lactobacilli* in intestinal contents, without strain-specific molecular identification, such as strain-targeted PCR or sequencing. We cannot fully distinguish exogenous *L. plantarum* DC2 from indigenous murine *Lactobacillus* species. Thus, the elevated total *Lactobacillus* counts in the treatment group may only partially originate from transiently surviving DC2. Second, the 28-day continuous gavage design lacked posttreatment sampling time points. Without monitoring the abundance of the strain after its administration was withdrawn, we were unable to confirm the persistent intestinal colonization of *L. plantarum* DC2. All observed bacterial increases merely represent temporary gastrointestinal residence during intervention. Third, 97% OTU clustering was adopted for 16S rRNA sequencing analysis because of early completion of raw data processing, whereas ASV pipelines provide higher strain-level resolution. The OTU-based results may underestimate fine-scale taxonomic variations. Fourth, the functional predictions of PICRUSt2 are purely bioinformatic in silico outputs rather than experimentally verified metabolic or immune activities, and all inferred pathway shifts require further laboratory validation. We therefore adopted cautious descriptive language for all microbial differences. Fifth, our histological evaluation was limited to villus height and crypt depth in healthy mice, and standardized inflammatory infiltration scoring or detection of tight junction proteins, inflammatory cytokines and intestinal permeability markers was not performed, which restricts comprehensive assessment of intestinal barrier function. Sixth, no commercial probiotic positive control group was established to benchmark the performance of *L. plantarum* DC2, so we cannot compare its regulatory effects with those of widely used reference strains. Finally, the mild reduction in weight gain observed in juvenile mice cannot be attributed to weight-regulating metabolism, as we lacked food intake monitoring, body composition and related metabolic indicators. Future research will address these limitations by adding strain-specific molecular tracing, postintervention sampling, ASV-based reanalysis, multiple functional intestinal biomarkers and reference probiotic controls to further validate the intestinal regulatory capacity of lyophilized *L. plantarum* DC2 powder.

## Conclusion

In this study, we established an optimal lyoprotectant formula and lyophilization workflow for producing Lactiplantibacillus plantarum DC2 powder, which effectively sustains strain viability and long-term storage stability. Supplementation with the lyophilized DC2 powder triggered distinct changes in intestinal morphological indices and gut microbial community structure in healthy mice. Taken together, these data reveal that lyophilized *L. plantarum* DC2 powder possesses the capacity to modulate intestinal morphology and gut microbiota profiles under healthy murine conditions. Follow-up experiments using intestinal disease animal models and human intervention trials are required to further verify its modulatory effects across broader research scenarios.

## Data Availability

The datasets presented in this study can be found in online repositories. The names of the repository/repositories and accession number(s) can be found at: https://www.ncbi.nlm.nih.gov/, SRA: SUB15310790; BioProject: PRJNA1261040.
